# Social Culture for Dentists

**Published:** 1888-03

**Authors:** W. F. Arnold

**Affiliations:** Rochester, N. Y.


### SOCIAL CULTURE FOR DENTISTS.

READ BEFORE A UNION MEETING OF THE 6TH, 7TH AND 8TH DISTRICT DENTAL SOCIETIES HELD IN BUFFALO, OCTOBER, 1887.

BY DR. W. F. ARNOLD, ROCHESTER, N. Y.

Every man who starts out in a professional career desires to be successful. To attain this end many qualities of mind and heart are needed. Good work and careful training in the various departments of the profession are not sufficient in themselves to give the full measure of success, though they are very important factors. What is needed is the rounding out of a man's social nature, and this can only come by careful culture. We owe to society a debt that must be paid, if we expect to collect our debts from society. This being the case, is it not a wise provision on your part to court and win the good-will and favor of a power so potent in its ability to pave the way to fortune and prosperity, or to sink you in hopeless ruin and adversity?

You may ask, how can this be accomplished? I answer, first, by being worthy; second, by showing your worth, as nothing tells like good work honestly and well done; third, by cultivating the social part of your nature, by smoothing off the rough corners, and by polishing up your intellect, manners and general address. It is well to have a few rules for every-day use in the matter of social culture.

*First.* Let no day pass without adding some new fact or thought to your store of information.

*Second.* Inform yourself as to the best usages of good society.

*Third.* Scrupulously keep this code of ethics.

*Fourth.* Attend strictly to your own affairs and guard against meddling with those of others.

*Fifth.* Choose good society and reject any that falls below your standard of right.

With these rules, go forth to create for yourself a place and name in the ranks where you would like to be known. The dentist should be entitled to enter the best circles of society, and he is, when he has complied with the requirements of their code. You will not find the doors to any circle open all at once. It takes time, and depends upon the tact of the person seeking admission through certain forms and legitimate introductions.

Man belongs to the gregarious type of animals, and when left alone becomes eccentric and odd, and exhibits many of the selfish traits of character seen in the lower orders of the brute creation. Look at the man who confines himself to his counting room and business entirely, and it will not be long before you will see some of the characteristics of the hog family developed in him.

The three great divisions of the social life are home, general society, and professional or business associations. The social home life should be the highest, the sweetest and dearest to every American heart. Here the tender love, the generous sympathy, the happy greeting, the sweet smiles of friends and family make the soundest institutions of our country. Here should be the cradle where the happiest manners are born, the truest and best friendships formed, where the kindest words are spoken and the most done to bring the ideal life of the best society to perfection. Without this effort at home to subdue your irritable and selfish dispositions, your future successes in society will be only shams of the most hypocritical order. Home is the foundation of our American institutions, and when the home-life becomes less sound, sweet and attractive, you may be sure there is social rottenness, and it will break out in social ulcers, which will sap all true social life. The two dearest words to Americans are Home and Mother; I count the moral downfall of any man from the day he repudiates these holy words, whether it be the home of his own mother or that of the mother of his children.

Society life is next to the home life. Every one needs some relaxation after the racking brain or muscular work of each day. I do not mean, when I recommend society, that you, as professional gentlemen, shall follow all the frivolities of fashionable life. You cannot afford the time or expenditure necessary to meet such demands. But I mean, when I recommend society for culture, that it shall be among good, sensible people, who recognize the fact that the night was made for sleep. This class is the thinking class, the reading class, the intelligent class, that make men sound, sweet and strong. These are the people who move the world and hold it in equilibrium. This circle comprises the busy people, who are working in churches, in reforms of all kinds, in the sciences and arts, and the broad fields of literature and education. They are the people who are living to some purpose, and in this class I recommend you to find your friends.

The claim has been made that dentists are, as a class, not very social among themselves—that they are kept apart by petty local jealousies and animosities, really beneath the notice of cultured gentlemen. If these conditions really do exist, no time should be lost in trying to heal old wounds and dress old sores till all are well. Then it may be said of us, as it is said of the societies of physicians, that we professionally consult together and help each other in difficult cases with right good-will.

I am pleased to say from my observation, and from what I have gathered from experience of others, that the broad liberal spirit of good-will and brotherly feeling is rapidly spreading among us, and has been brought about by our frequent meetings in the dental circles. I have been glad to see the growing respect and dignity manifested. Men have learned that no good, substantial structure is reared on the ruin of another. The way to grow socially, as dentists among ourselves, is to attend the city, district and State society meetings. In these we are brought together to contribute our mite to the general good. We get new ideas and are better men for this intercourse. Our societies are elevating in their tendencies, and are growing more and more into dignified, useful institutions. The greatest evil to guard against is the fondness for self-display, and the greed for official position. I could not help feeling, when I attended a great Dental Association, that the principal question seemed to be, for whom are you going to vote? As if the Association was only a body of wire pullers, and its principal object the election of some one to office!

Our social dental clubs, I believe, can be made very useful to promote a higher educational standard and culture among us, provided they do not entirely lose sight of their object, and degenerate into mere drinking and smoking clubs, where the best fellow is he who can tell the most interesting story. The dental club that is a working institution, where papers are carefully prepared and read, where difficult pathological cases are discussed, where matters of interest to the profession are considered, with enough of literature, and perhaps music introduced to make the evening intructive, is a blessing to all who are its members. Our profession is most honorable and useful, and what it needs most is men who honor it by bringing to it a good preparation and a broad, liberal education and culture.

